# Reduced risk of skin cancer and internal malignancies in vitiligo patients: a retrospective population-based cohort study in Taiwan

**DOI:** 10.1038/s41598-021-99786-9

**Published:** 2021-10-12

**Authors:** Yu-Ching Weng, Hsiu J. Ho, Yi-Ling Chang, Yun-Ting Chang, Chun-Ying Wu, Yi-Ju Chen

**Affiliations:** 1 Department of Dermatology, Taichung Veterans General Hospital, No. 1650, Sec. 4, Taiwan Blvd., Taichung, 407 Taiwan; 2 Institute of Biomedical Informatics, Institute of Public Health, National Yang-Ming University, No. 155, Section 2, Linong Street, Taipei, 11221 Taiwan; 3 Faculty of Medicine and Institute of Clinical Medicine, School of Medicine, National Yang-Ming University, Taipei, Taiwan; 4 Department of Dermatology, Taipei Veterans General Hospital, Taipei, Taiwan; 5 Division of Translational Research and Center of Excellence for Cancer Research, Taipei Veterans General Hospital, Taipei, Taiwan; 6 Department of Public Health, China Medical University, Taichung, Taiwan; 7 National Institute of Cancer Research, National Health Research Institutes, Miaoli, Taiwan

**Keywords:** Cancer, Medical research, Oncology

## Abstract

The relationship between cancer and vitiligo has been explored but with inconsistent results. To examine the long-term cancer risk in vitiligo patients, we conducted a retrospective nationwide cohort study. From the National Health Insurance Research Database of Taiwan, a total of 13,824 vitiligo patients were identified and matched with 55,296 reference subjects without vitiligo by age, gender, and propensity score estimated by major comorbidities from 1997 to 2013. Demographic characteristics and comorbidities were compared between these two groups. Incidence rate ratios and hazard ratios (HRs) were calculated to examine cancer risks. The 16-year incidence rates of overall cancers were 621.06 (566.56–675.55) and 726.99 (697.24–756.74) per 100,000 person-years in the vitiligo and reference groups. Patients with vitiligo showed a significantly decreased risk of overall cancers [adjusted HR, 0.85; 95% confidence interval (CI), 0.77 to 0.93, *p* < 0.001] compared with reference subjects without vitiligo after adjusting for age, sex, comorbidities, and treatments. The risks of basal cell carcinoma (BCC) and squamous cell carcinoma (SCC) were significantly reduced (adjusted HR 0.21, 95% CI 0.11–0.38, *p* < 0.001), as well as internal malignancies (adjusted HR 0.89, 95% CI 0.81–0.99, *p* = 0.026). The results were consistent across different subgroups of patients, including male gender, ages more than 40 years, and those receiving long-term systemic disease-modifying antirheumatic drugs and phototherapies. Information related to phenotype, disease duration, vitiligo lesion sites, family history of vitiligo or cancer, occupation, and personal lifestyle was not included in the database. Vitiligo is associated with reduced risks of BCC and SCC, as well as internal malignancies.

## Introduction

The prevalence of vitiligo worldwide is 0.4–2%^1^. It is an autoimmune disease that destroys melanocytes of the skin and occurs in all age groups, causing devastating psychological effects^2^. Many studies have pointed to an immune system association in vitiligo, such as circulating CD8 + cytotoxic T-lymphocytes (CTL), increased expression of interferon-γ (IFN-γ) in lesional skin, and increased programmed death ligand-1 (PD-L1) in regulatory T cells (Treg)^2–4^.

The associations of vitiligo with melanoma and non-melanoma skin cancer (NMSC)^5–18^, and internal malignancies^19,20^ have been explored. Carcinogenesis has long been a significant concern in using long-term repetitive phototherapy^21^ or systemic disease-modifying antirheumatic drugs (DMARDs)^22^ for unstable and progressive vitiligo. However, an increasing number of studies have demonstrated that an enhanced immune system suppresses cancer and increases tumor cell surveillance^10–13^. Markedly reduced risks of internal malignancies in patients with vitiligo, such as colon and rectal cancers, lung cancer, and ovarian cancer, as well as an increased risk for melanoma and non-melanoma skin cancer (NMSC) have been recently reported in two nationwide cohort studies in Korea^17,19^. However, Li et al. reported higher risks of thyroid and breast cancers in female vitiligo patients and bladder cancer in both genders of vitiligo patients in a nationwide cohort study in Taiwan^20^.

In this study, we aimed to verify the long-term risk of cancers in vitiligo patients. Patients with vitiligo and age-, gender-, and propensity score^23^-matched reference subjects were included for comparison. The effect of phototherapy and systemic DMARDs were also evaluated.

## Materials and methods

### Study design

We performed a nationwide cohort study by including patients diagnosed with vitiligo and age-, gender-, and propensity score-matched control subjects from Taiwan's National Health Insurance Research Database (NHIRD) period 1997–2013. The NHIRD is a public database provided by the National Health Insurance Administration, Ministry of Health and Welfare, Taiwan. NHIRD has been validated^24^ and utilized extensively in epidemiologic studies in Taiwan (https://nhird.nhir.org.tw/en)^25–27^. In this database, the diagnostic codes are in the International Classification of Diseases format, Revision 9, Clinical Modification (ICD-9-CM), with diagnoses made by board-certified physicians in the corresponding specialties. Personal information, including body weight, height, family history, laboratory examination results, lifestyle, and habits such as smoking and alcohol use, is not available from the NHIRD.

This study has been approved by the Institutional Review Board of Taipei Veterans General Hospital, Taipei, Taiwan (No. 2017-08-005CC).

### Participants and matched reference subjects

All patients with a primary diagnosis of vitiligo (ICD-9-CM code 709.01) for the first time between 1997 and 2013 were eligible for this study. We included only those subjects who had received a major diagnosis of vitiligo more than three times in an outpatient department or at least one time in an inpatient department by dermatologists. Subjects with diagnostic codes for diseases mimicking vitiligo, including pityriasis versicolor (ICD-9-CM code 111.0), pityriasis alba (ICD-9-CM code 696.5), and morphea (ICD-9-CM codes 701.0), were excluded to prevent misdiagnosis or uncertain diagnosis of vitiligo. Subjects with dubious basic data, such as conflicting gender or uncertain birth date or prior history of cancer, were also excluded. Reference subjects without a history of vitiligo or cancer were randomly selected from NHIRD. Study subjects in both groups who had medical visits every year during the observation period were selected for further analysis.

We matched each subject in the vitiligo group with four reference subjects without vitiligo by age (on birth year), gender, and propensity score^23^. We performed propensity score (PS) matching to reduce confounders in observational studies by using the nearest neighbor algorithm with a 1:4 ratio, without replacement, and a caliper width of 0.0001. Logistic regression analysis was performed to calculate PS in a logistic model. Covariates such as acute coronary syndrome, diabetes mellitus, hypertension, chronic liver disease, chronic renal disease, autoimmune diseases during the observation time were included in the propensity score model. The standardized difference used to assess covariate balance^28^ between the two groups was less than 0.1.

A total of 13,824 vitiligo patients and 55,296 matched reference subjects were enrolled. Among vitiligo patients, 27 patients had received the diagnosis when they were admitted for other skin diseases. The distribution of each age group was presented in Table 1.

**Table 1:** Demographic characteristics of vitiligo and reference subjects after matching by age, gender and propensity score.

Characteristics	Vitiligo cohort		Reference cohort		Standardized difference	*p* value
	(N = 13,824)		(N = 55,296)			
	Number	%	Number	%		
**Age, years**					0.000	
Mean ± SD	43.5 ± 20.5		43.5 ± 20.5			0.994
Median (Q1-Q3)	44.7 (27.6–58.5)		44.8 (27.7–58.6)			0.996
< 20	2176	15.7	8680	15.7		
20–39	3721	26.9	14,864	26.9		
40–59	4774	34.5	19,095	34.5		
≥ 60	3153	22.8	12,657	22.9		
**Hospital visits, N**						
Mean ± SD	155.9.3 ± 135.4		134.2 ± 128.9		0.164	< 0.001
Medium (Q1-Q3)	118.0 (65.0–204.0)		96.0 (48.0–178.0)			< 0.001
**Follow-up, yrs**					0.042	
Mean ± SD	5.8 ± 2.5		5.7 ± 2.5			< 0.001
Median (Q1-Q3)	5.9 (3.6–7.9)		5.6 (3.6–7.8)			< 0.001
**Gender**					0.000	> 0.999
Female	8025	58.1	32,100	58.1		
Male	5799	41.9	23,196	41.9		
**Comorbidities**						
ACS	1303	9.4	5222	9.4	0.001	0.961
DM	1090	7.9	4372	7.9	0.001	0.947
Hypertension	2698	19.5	10,880	19.7	0.004	0.682
Chronic liver disease	1795	13.0	7107	12.9	0.004	0.689
Chronic renal disease	132	1.0	528	1.0	0.000	> 0.999
Autoimmune diseases^a^	184	1.3	736	1.3	0.000	> 0.999
RA	81	0.6	418	0.8	0.021	0.040
SLE	45	0.3	167	0.3	0.004	0.718
SS	71	0.5	176	0.3	0.03	< 0.001
Dermatomyositis	3	0.0	13	0.0	0.001	> 0.999
Phototherapy^b^	876	6.34	77	0.14	0.356	< 0.001
DMARDs^b^	268	1.9	1007	1.8	0.009	0.377

*SD* standard deviation, *yrs* years, *ACS* acute coronary syndrome, *BCC* basal cell carcinoma, *DM* diabetes mellitus, *DMARDs* disease modifying antirheumatic drugs, *N* number, *RA* rheumatoid arthritis, *SLE* systemic lupus erythematosus, *SS* sicca syndrome, *SCC* squamous cell carcinoma.

Propensity score matching on the factors including ACS, DM, hypertension, chronic liver disease, chronic renal diseases and autoimmune diseases.

^a^Autoimmune diseases include RA, SLE, SS, and dermatomyositis, all of which are registered in the catastrophic illness dataset.

^b^Phototherapy indicated receiving phototherapy including ultraviolet B (UVB) or psoralen-UVA for more than 100 sessions during observation time. Long term DMARDs indicated using systemic DMARDs for more than 30 days on average per year of observation.

The date of the first vitiligo diagnosis or the corresponding date of the matched reference subjects was defined as the index date. All sampled individuals were followed up until 31 Dec. 2013 (the end of the dataset follow-ups), the outcome of interest (i.e., the earliest time of cancer diagnosis), registration of the death, or withdrawal from Taiwan's National Health Insurance program.

### Outcome measurement

The disease of outcome was any cancer event. We validated the diagnoses of cancers with records from the Registry of Catastrophic Illness Patient Database, a separate subpart of the NHIRD. Insured patients who suffer from major diseases, such as malignancy, can apply for a catastrophic illness certificate, which grants exemption from all co-payments. Applications for catastrophic illness certificates are validated by at least two specialists based on careful examination of medical records, laboratory data, and imaging studies. Cytological or pathological reports or other evidence supporting the diagnosis is also required. Carcinoma in situ was not included. Only those patients meeting the diagnostic criteria of major diseases are issued a catastrophic illness certificate. The high level of accuracy of the Registry of Catastrophic Illness Patient Database in validating cancer diagnoses has been verified in previous studies^29^. The diagnostic codes of malignancies were defined as those from 140 to 208 in the ICD-9-CM format. Outpatient and inpatient claims by beneficiaries with a registered catastrophic illness are collected in the catastrophic illness profile and distributed as a package.

### Confounding factors

Demographic factors, such as age, gender, and propensity score constructed by comorbidities mentioned above, were considered potential confounders. Comorbid diseases were included if diagnosed at least three times.

Patients with rheumatoid arthritis (RA), systemic lupus erythematosus (SLE), sicca syndrome (SS), and dermatomyositis, were identified from the Registry of Catastrophic Illness Patient Database.

### Cancer risk analysis

All enrolled patients were followed up from the index date of the first diagnosis of vitiligo or the corresponding dates of non-vitiligo reference subjects, until the earliest time of cancer diagnosis, death, the end of follow-up in the personal medical records, or 31 December 2013 (i.e. the end of dataset follow-up). The number of specific cancer types and incidence rate ratios (IRRs) of primary cancers was calculated, and stratified analyses were conducted.

### Statistical analysis

The demographic data of the study population was first analyzed. We compared the demographic factors and prevalence of disease outcomes between the two study cohorts by chi-square test. To assess the age and sex effects on relative risks of malignancies, we categorized all enrollees by sex and ages 20–39 years, 40–59 years, and ≥ 60 years at the first diagnosis of vitiligo. Phototherapy (including ultraviolet B (UVB) and psoralen plus ultraviolet A [PUVA]) and long-term systemic DMARDs for vitiligo was considered a proxy for extensive or active vitiligo disease. DMARDs, including methotrexate, cyclosporine, and azathioprine, were analyzed. Exposure to these drugs was defined as a duration of use of more than 90 days during the observation period. We, therefore, conducted a posthoc subgroup analysis based on different treatment modalities. We examined the risks of specific cancer types among vitiligo patients using the incidence rate (IR). IR was estimated as the number of cancer cases among study patients divided by the sum of time at risk, i.e., the number of person-years. The IRR and 95% confidence intervals (CI) were calculated as the ratio of IRs in exposed (vitiligo) and unexposed (non-vitiligo) individuals using natural logarithm estimates.

We further conducted multivariate analyses on each specific cancer type and different treatment groups under the Cox proportional hazards model after adjusting for age, gender, and comorbidities, to ensure our results' consistency. The adjusted hazard ratios (HRs) and 95% CI were calculated.

Data were managed with SAS 10.4 software (SAS Institute Inc., Cary, NC, USA). And the "cmprsk" package of R (http://cran.r-project.org/web/packages/cmprsk/index.html). Calculated results are expressed as the estimated number together with 95% CI.

## Results

### Demographic characteristics and cumulative incidences of cancers

**Figure 1:**
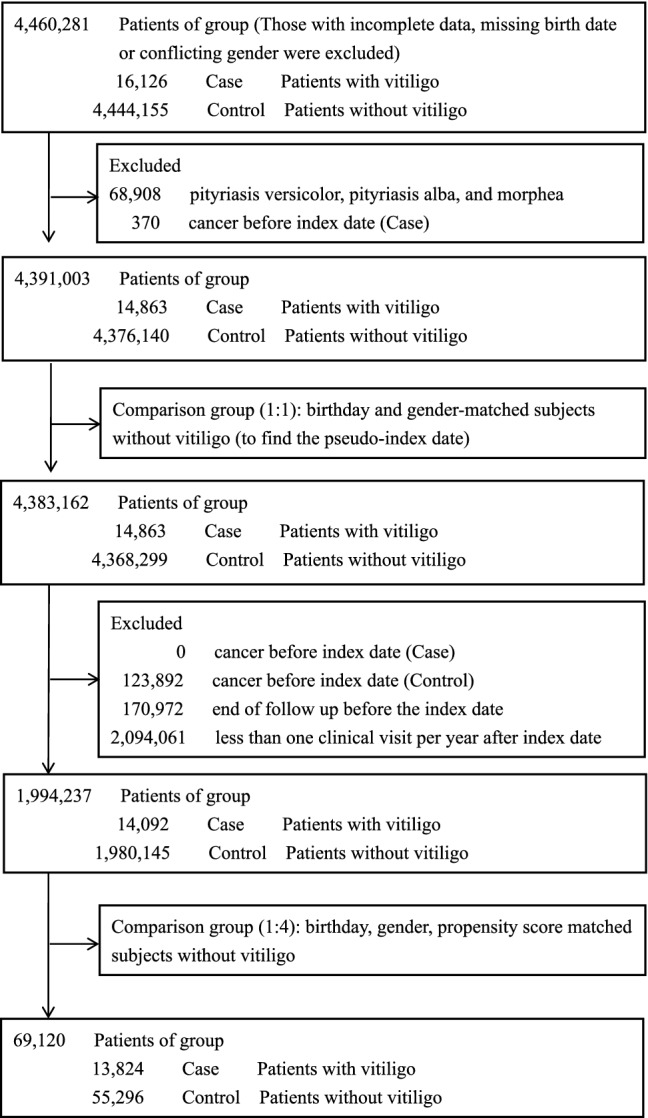
Selection process for subjects in vitiligo and control cohorts.

Subjects in both study cohorts were selected according to the process presented in Fig. 1. Demographic characteristics and associated comorbidities are shown in Table 1. A total of 499 patients (3.6%) with vitiligo and 2294 patients (4.1%) without vitiligo developed cancer (*p* = 0.004). The mean follow-up times were 5.8 ± 2.5 years in patients with vitiligo and 5.7 ± 2.5 years in patients without vitiligo. The prevalence of major comorbidities was comparable between the groups after matching on the age, gender and propensity score (Table 1).

Among vitiligo patients, 3761 patients (27.2%) had received phototherapies for at least ten sessions. Only 876 (6.34%) received more than 100 sessions of phototherapies during the observation time. A total of 268 vitiligo patients (1.9%) had received long-term DMARDs. These patients were considered to have an extensive or active vitiligo disease.

### Incidence rates and adjusted hazard ratios of overall cancer and specific cancer types

The IR for overall cancer in vitiligo patients was 621.06 (566.56–675.55) per 100,000 person-years. The relative risk of overall cancer in patients with vitiligo was significantly lower than that in patients without vitiligo (adjusted HR [aHR] 0.82, 95% CI 0.74–0.90, *p* < 0.001) (Table 2, eTable 1).

**Table 2:** Incidence rates and adjusted hazard ratios of cancer risk between vitiligo patients and reference subjects.

	Patients With Vitiligo (N = 13,824)			Controls Without Vitiligo (N = 55,296)			Multivariate analysis	
	IR (95% CI)^a^	Events	PY	IR (95% CI)^a^	Events	PY	aHR (95% CI)^b^	*P*-value
Cancer, overall	621.06 (566.56–675.55)	499	80,347.09	726.99 (697.24–756.74)	2294	315,547.23	0.82 (0.74–0.90)	< 0.001
Oral cavity, and pharynx	30.74 (18.69–42.79)	25	81,336.64	35.59 (29.05–42.12)	114	320,358.05	0.87 (0.56–1.35)	0.538
Thyroid	22.13 (11.90–32.35)	18	81,351.38	17.16 (12.63–21.70)	55	320,448.03	1.24 (0.73–2.12)	0.422
Digestive system^c^	230.64 (197.58–263.70)	187	81,077.74	253.85 (236.37–271.34)	810	319,079.84	0.86 (0.73–1.01)	0.066
Stomach	29.50 (17.70–41.31)	24	81,350.07	28.09 (22.28–33.89)	90	320,434.53	0.96 (0.60–1.53)	0.862
Colon, Rectum	87.35 (67.03–107.66)	71	81,286.76	89.98 (79.59–100.37)	288	320,074.28	0.96 (0.74–1.24)	0.735
Liver	88.61 (68.14–109.08)	72	81,255.49	100.36 (89.38–111.34)	321	319,859.86	0.80 (0.61–1.04)	0.095
Pancreas	9.83 (3.02–16.64)	8	81,395.03	12.17 (8.35–15.98)	39	320,562.62	0.81 (0.38–1.74)	0.588
Gall bladder	7.37 (1.47–13.27)	6	81,383.86	7.49 (4.49–10.48)	24	320,573.19	0.83 (0.32–2.15)	0.709
Other	25.81 (14.77–36.84)	21	81,374.69	36.20 (29.61–42.78)	116	320,467.03	0.71 (0.44–1.14)	0.153
Respiratory system^c^	86.11 (65.94–106.28)	70	81,292.41	104.03 (92.86–115.20)	333	320,099.73	0.84 (0.64–1.08)	0.176
Lung, pleura	75.01 (56.19–93.84)	61	81,321.43	94.01 (83.39–104.63)	301	320,186.26	0.81 (0.61–1.06)	0.128
Other	11.06 (3.83–18.29)	9	81,367.41	10.30 (6.78–13.81)	33	320,496.97	1.02 (0.49–2.15)	0.949
Skin and others^c^	103.48 (81.35–125.60)	84	81,718.81	156.10 (142.39–169.81)	498	319,029.79	0.63 (0.50–0.80)	< 0.001
MM	1.23 (0–3.64)	1	81,396.00	8.42 (5.25–11.60)	27	320,529.82	0.14 (0.02–1.07)	0.058
SCC, BCC	13.52 (5.53–21.51)	11	81,378.35	60.96 (52.40–69.52)	195	319,884.67	0.21 (0.11–0.38)	< 0.001
Female breast	82.51 (62.75–102.26)	67	81,204.56	79.10 (69.35–88.85)	253	319,841.07	1.00 (0.76–1.32)	0.977
Other	6.14 (0.76–11.53)	5	81,389.17	8.42 (5.25–11.60)	27	320,528.07	0.59 (0.21–1.62)	0.307
GU system^c^	130.68 (105.80–155.55)	106	81,116.20	124.52 (112.28–136.75)	398	319,639.63	0.99 (0.79–1.23)	0.907
Uterus	25.82 (14.78–36.87)	21	81,320.84	37.16 (30.48–43.83)	119	320,265.06	0.68 (0.42–1.09)	0.108
Cervix	8.60 (2.23–14.98)	7	81,366.16	10.61 (7.04–14.17)	34	320,487.41	0.76 (0.34–1.71)	0.507
Uterus	1.23 (0–3.64)	1	81,396.00	8.74 (5.50–11.97)	28	320,524.19	0.14 (0.02–1.03)	0.053
Ovary	9.83 (3.02–16.65)	8	81,361.40	11.54 (7.82–15.26)	37	320,501.78	0.84 (0.39–1.80)	0.653
Testis	NA	0	81,396.42	2.18 (0.57–3.80)	7	320,564.86	NA	NA
Bladder	30.74 (18.69–42.79)	25	81,329.29	28.72 (22.85–34.58)	92	320,375.94	0.91 (0.58–1.44)	0.701
Prostate	54.13 (38.14–70.12)	44	81,285.65	38.09 (31.33–44.85)	122	320,301.38	1.27 (0.90–1.79)	0.182
Kidney	22.12 (11.90–32.34)	18	81,367.01	22.47 (17.28–27.66)	72	320,444.74	0.89 (0.53–1.50)	0.665
Others	51.66 (36.04–67.29)	42	81,296.88	49.34 (41.64–57.03)	158	320,245.88	0.94 (0.67–1.33)	0.737
Hematopoietic system^c^	29.50 (17.70–41.30)	24	81,359.28	38.39 (31.61–45.18)	123	320,357.44	0.75 (0.48–1.17)	0.204
Lymphoma	13.52 (5.53–21.51)	11	81,373.40	20.60 (15.63–25.56)	66	320,453.95	0.62 (0.32–1.20)	0.158
Leukemia	12.29 (4.67–19.90)	10	81,384.48	12.17 (8.35–15.99)	39	320,528.41	0.96 (0.47–1.97)	0.911
Multiple myeloma	3.69 (0–7.86)	3	81,394.23	4.99 (2.55–7.44)	16	320,559.13	0.77 (0.22–2.66)	0.682
Hodgkin's disease	NA	0	81,396.42	1.56 (0.19–2.93)	5	320,569.42	NA	NA
Unspecified^c^	38.12 (24.70–51.54)	31	81,328.12	33.40 (27.07–39.73)	107	320,375.60	1.16 (0.77–1.74)	0.474
All except skin cancers	605.98 (552.16–659.80)	487	80,365.59	655.36 (627.14–683.57)	2,073	316,317.11	0.89 (0.81–0.99)	0.026

*BCC* basal cell carcinoma, *CI* confidence intervals, *HR* hazard ratio, *IR* incidence rates, *MM* malignant melanoma, *PY* person-years, *SCC* squamous cell carcinoma.

^a^Incidence rate per 100,000 person-years.

^b^Adjustment for age, gender, hospital visits, and comorbidities listed in Table 1.

^c^Digestive system includes gastrointestinal system, liver, spleen, gall bladder. Respiratory system includes respiratory and intrathroax organs. Skin and others include skin, bone, connective tissue, and breast. GU system includes genital organs: prostate, scrotum, and urinary tract, as well as uterus, cervix, and ovaries. Hematopoietic system includes leukemia, lymphoma, and other hematologic cancers. Unspecified include other malignant solid tumors not mentioned above.

Risks of NMSC, including basal cell carcinoma (BCC) and squamous cell carcinoma (SCC) were significantly reduced with aHR of 0.21 (95% CI 0.11–0.38, *p* < 0.001). There is a lower risk of malignant melanoma (MM) in vitiligo patients, yet without statistical significance (aHR 0.14, 95% CI 0.12–1.07, *p* = 0.058). After excluding NMSC and melanoma, the risk of internal malignancies remained reduced (aHR 0.89, 95% CI 0.81–0.99, *p* = 0.026).

On the contrary, the risks of prostate cancer and thyroid cancer were increased, yet without statistical significance on multivariate analysis. There were five (27.8%) of hyperthyroidism/thyroiditis and one (5.56%) of hypothyroidism preceding thyroid cancer patients in vitiligo group, compared to nine (16.36%) and three (5.45%), respectively, in non-vitiligo reference subjects.

The relative risk of cancer in vitiligo patients was consistently reduced in different ages and gender. Cancer risk of vitiligo patients significantly decreased in males (IRR 0.82, 95% CI 0.72–0.95, *p* = 0.006) and those aged 40–59 (IRR 0.76, 95% CI 0.64–0.90, *P*-value 0.002) and ≥ 60 (IRR 0.85, 95% CI 0.75–0.96, *P* = 0.011). The reduced risk of cancer was consistent among those receiving long-term phototherapies or DMARDs (eTable 1).

### Multivariate analysis

Vitiligo was independently associated with reduced cancer risk (aHR 0.85, 95% CI 0.77–0.93, *p* < 0.001) after adjusting for age, gender, study groups, and comorbidities. Other independent risk factors included increasing age and males. Long-term phototherapy exposures and long-term DAMRDs were not associated with cancer risk **(**Table 3).

**Table 3:** Multivariate analysis of predicting factors for cancers among study cohorts.

	N	Cancer	HR (95%CI)	*p*-value	aHR (95%CI)^a^	*p*-value
Age	–	–	1.05 (1.05–1.06)	< 0.001	1.05 (1.05–1.05)	< 0.001
Male	28,995	1376	1.36 (1.26–1.46)	< 0.001	1.21 (1.12–1.30)	< 0.001
Vitiligo	13,824	499	0.86 (0.78–0.95)	0.003	0.85 (0.77–0.93)	< 0.001
Phototherapy^b^	953	26	0.59 (0.40–0.86)	0.007	0.76 (0.52–1.12)	0.172
DMARDs^b^	1275	53	0.98 (0.75–1.28)	0.884	0.90 (0.68–1.19)	0.465

*aHR* adjusted hazard ratio, *DMARDs* disease modifying antirheumatic drugs, *HR* hazard ratio, *N* case number.

^a^Adjustment for age, gender, study groups, hospital visit number, long term exposure of phototherapy and use of DMARDs, and major comorbidities including cardiovascular diseases, chronic liver disease, chronic renal diseases, and autoimmune diseases.

^b^Indicates receiving long term ultraviolet B or ultraviolet A phototherapy for more than 100 sessions during the observation time. Use of DMARDs for more than 30 days per year on average during the observation time.

## Discussion

The results of this 16-year nationwide cohort study suggested reduced risks of overall cancers in vitiligo patients, especially BCC and SCC, compared to matched non-vitiligo reference subjects. In addition to increasing age and males, vitiligo was independently associated with a lower cancer risk on multivariate analysis. Long-term DMARDs or phototherapies were not associated with cancer risk.

The relationship between vitiligo and malignancies has been debated. A few case reports and studies have suggested an association between vitiligo and internal malignancies^30–32^. Large-scale nationwide cohort studies of cancer risk in vitiligo patients have been rare^17–20^. Bae et al. reported markedly reduced risks of internal malignancies in patients with vitiligo (hazard ratio [HR] 0.86), especially colon and rectal cancer (HR 0.62), lung cancer (HR 0.75), and ovarian cancer (HR 0.62)^19^. Li et al. earlier has demonstrated increased risks of the thyroid (standardized incidence ratio [SIR] 3.39) in vitiligo patients^20^, which is consistent with results from Bae’s study and the current study. The former compared cancer risk between vitiligo patients and age-, gender-, and major comorbidities-matched non-vitiligo subjects. The latter estimated cancer risk associated with vitiligo based on age- and gender-standardized cancer registry, without matching comorbidities. Vitiligo has been associated with autoimmune diseases, such as autoimmune thyroid disease, type 1 diabetes mellitus, rheumatoid arthritis, and SLE, which may increase cancer risk^33^. The increased risk of cancer in vitiligo patients in Li's study might be attributed to the coexistence of comorbidities among vitiligo patients. After matching these major comorbidities, the current study's results consistently revealed a reduced risk of overall cancers in vitiligo patients, mainly for BCC and SCC.

The risks of many internal malignancies were reduced in our vitiligo patients, however, without statistical significance. The different results from Bae's study may be partly due to the relatively smaller number of study subjects and the limited number of cancer events in the current study, resulting in underpowered estimates. Compared to Bae's study, we have a lower incidence rate of internal malignancies in the patients without vitiligo, for example, a marked lower incidence rates of stomach and colorectal cancers; and a higher incidence rate of liver and lung cancers. The relative risk of cancer for vitiligo could be very different^19,20^.

The decreased risk of NMSC, including SCC and BCC, in vitiligo patients, has been widely explored^10–13^. One possible reason for this association is that vitiligo patients receive instructions on protecting their skin from the sun^6,8,11^. An overexpression of epidermal wild-type p53 and Mdm2, which is a negative regulator of p53, has been observed in vitiligo patients compared to NMSC patients^33^. Overexpression of p53 tumor suppressor gene^9,11,34^ and overproduction of pro-inflammatory cytokines (interleukin-1 and tumor necrosis factor alpha) have been proposed to stimulate the production of glutathione peroxidase and superoxide dismutase, resulting in reduced risk of skin cancer and anti-melanocyte immune response in vitiligo patients^35^. Contrarily, Kim et al.^17^ reported an increased risk of melanoma and NMSC in vitiligo patients, which was conflict from results of the literatures^6–16,19^ and the current study. The reasons for the conflicting results remained to be clarified. Here we conducted further sensitivity analysis and demonstrated consistent negative associations between vitiligo and cancers, even in those receiving long-term phototherapies or DMARDs (Table 3, eTable 1).

Long-term phototherapy has been a concern for its potential skin cancer risk. We previously reported that long-term narrowband (NB)-UVB phototherapy does not increase skin cancer risk than short-term NB-UVB phototherapy in a 13-year nationwide cohort study^36^. Teulings et al. demonstrated that age-adjusted melanoma and NMSC lifetime prevalence do not grow in patients treated with phototherapy, including NB-UVB and PUVA, no matter the number of treatment sessions^9^. One recent nationwide population-based retrospective cohort study demonstrated that long-term NB-UVB, up to 500 sessions, does not increase NMSC risk in Korean vitiligo patients^37^. The results were consistent with our current study. Long-term NB-UVB phototherapy is considered a safe treatment for vitiligo.

The results of this study revealed an increased risk of prostate cancer, yet without statistical significance. Vitamin D metabolites have been reported to have pro-differentiating and anti-proliferative effects on prostate cancer cell lines. Vitamin D deficiency has also been shown to be associated with an increased risk of prostate cancer^38^. Silverberg et al. reported that 55.6% of vitiligo patients are deficient in 25-hydroxyvitamin D^39^. Another 10-year retrospective study demonstrated that 57% of patients with vitiligo have an insufficient or low level of 25-OH vitamin D^40^. Therefore, vitamin D deficiency might be the link between vitiligo and prostate cancer. Further studies are needed to confirm this association.

The strength of this study is the utilization of Taiwan’s NHIRD, which contains comprehensive medical information for more than 99% of all residents in Taiwan. The large population of enrollees was sufficient for investigating cancer risk and avoiding selection bias.

However, there were several limitations. First, subjects in study cohorts may differ in measured and unmeasured ways. We did not have access to family history of vitiligo or cancer, diet, alcohol consumption, smoking habit, lifestyles such as occupational and leisure ultraviolet or sunlight exposure, or laboratory data. No detailed information on disease severity, duration, sites or type of vitiligo, or skin sites that have received phototherapy, was available. Therefore, we could not examine the effect of these factors on cancer risk in the present study. Instead, we selected subjects for the two study cohorts by matching age, gender, and propensity scores estimated by major comorbidities. We further conducted subgroup analysis by restricting those with long-term exposure to phototherapies or DMARDs, a proxy for more extensive or an active vitiligo disease, to ensure the consistency of study results. Second, misclassification bias may occur when identifying study subjects by diagnostic codes. Here, we included patients with vitiligo who had more than three diagnoses in an outpatient department or who had been admitted to a hospital by dermatologists. We excluded subjects diagnosed with pityriasis versicolor, pityriasis alba, or morphea, which might mimic vitiligo. The prevalence of vitiligo might have been underestimated. Yet, we, therefore, reduce the potential misclassification of study subjects. Finally, more hospital visits in study subjects may provide a greater chance for cancer detection. This potential surveillance bias may have led to an overestimation of cancer in vitiligo patients. Conversely, we found a lower risk of cancer in vitiligo patients. A possible underestimation of the negative association between vitiligo and cancer may occur.

In conclusion, this nationwide matched cohort study showed that vitiligo is associated with a reduced risk of skin cancers and internal malignancies.

## Supplementary Information

Supplementary Information.

## Data Availability

The data that support the findings of this study are available from the Bureau of National Health Insurance, Department of Health, and managed by National Health Research Institutes. Restrictions apply to the availability of these data, which were used under license for this study. Data are available with the permission of the National Health Research Institutes, Taiwan.
