# Supplementary material for: Reduced risk of skin cancer and internal malignancies in vitiligo patients: a retrospective population-based cohort study in Taiwan
Source: Sci Rep. 2021 Oct 12;11:20195. doi: 10.1038/s41598-021-99786-9 (PMC8511292; doi:10.1038/s41598-021-99786-9)
Supplement: Supplementary file 1 — Supplementary Information. [file 41598_2021_99786_MOESM1_ESM.docx]

|  | | Patients With Vitiligo (N=13,824) | | | | | | | | | Controls Without Vitiligo (N=55,296) | | | | | | | | | | | |  | | | | | |  |
| --- | --- | --- | --- | --- | --- | --- | --- | --- | --- | --- | --- | --- | --- | --- | --- | --- | --- | --- | --- | --- | --- | --- | --- | --- | --- | --- | --- | --- | --- |
|  | | IR (95% CI) | | | Events | | | PY | | IR (95% CI) | | | | | | Events | | | PY | | IRR (95% CI) | | | | P value | | | |  |
| **Sex** | |  | | |  | | |  | | | |  | | | |  | | |  | | |  | | | |  | | | |
| Female | | 549.73 (482.78-616.68) | 259 | | | 47,113.72 | | | | 622.90 (587.02-658.78) | | | 1,158 | | | | 185,904.90 | | | | 0.88 (0.77-1.01) | | | | 0.069 | | | |  |
| Male | | 722.17 (630.80-813.53) | 240 | | | 33,233.37 | | | | 876.26 (825.30-927.21) | | | 1,136 | | | | 129,642.33 | | | | 0.82 (0.72-0.95) | | | | 0.006 | | | |  |
| **Age, years** | |  |  | | |  | | | |  | | |  | | | |  | | | |  | | | |  | | | |  |
| 20-39 | | 165.04 (111.86-218.22) | 37 | | | 22,418.57 | | | | 147.86 (122.73-172.99) | | | 133 | | | | 89,948.42 | | | | 1.12 (0.78-1.61) | | | | 0.554 | | | |  |
| 40-59 | | 567.00 (478.87-655.14) | 159 | | | 28,042.16 | | | | 745.16 (694.25-796.07) | | | 823 | | | | 110,446.15 | | | | 0.76 (0.64-0.90) | | | | 0.002 | | | |  |
| ≥ 60 | | 1,745.29 (1,547.79-1,942.79) | 300 | | | 17,189.13 | | | | 2,053.31 (1,942.74-2,163.87) | | | 1,325 | | | | 64,530.11 | | | | 0.85 (0.75-0.96) | | | | 0.011 | | | |  |
| †Phototherapy, >500 | | 273.31 (-262.38-809.01) | 1 | | | 365.88 | | | | NA | | | 0 | | | | 61.17 | | | | NA | | | | NA | | | |  |
| †Phototherapy, >100 | | 359.06 (205.49-512.63) | 21 | | | 5,848.59 | | | | 1,030.57 (127.24-1,933.90) | | | 5 | | | | 485.17 | | | | 0.35 (0.13-0.92) | | | | 0.034 | | | |  |
| DMARDs* | | 205.73 (0-438.53) | 3 | | | 1,458.25 | | | | 813.25 (587.83-1038.67) | | | | 50 | | | 6,148.19 | | | | 0.25 (0.08-0.81) | | | | 0.021 | |  |  |  |
| DMARDs, all except skin | | 205.73 (0-438.53) | 3 | | | 1,458.25 | | | | 797.72 (574.36-1,021.09) | | | 49 | | | | 6,142.48 | | | | 0.26 (0.08-0.83) | | | | 0.023 | |  |  |  |

Supplementary Table 1. Risk of cancer in vitiligo and reference subjects, stratified by age, gender, use of phototherapies and DMARDs.

Abbreviations: CI, confidence intervals; IR, incidence rate; PY, person years. BCC, basal cell carcinoma; DM, diabetes mellitus; DMARDs, disease modifying antirheumatic drugs; N, number; RA, rheumatoid arthritis; SLE, systemic lupus erythematosus; SS, sicca syndrome; SCC, squamous cell carcinoma;

† Phototherapy indicated receiving phototherapy including ultraviolet B (UVB) or psoralen-UVA for more than 100 or 500 sessions during observation time.

*Long term DMARDs indicated using systemic DMARDs for more than 30 days on average per year of observation.
